# 3D Printing in Breast Reconstruction: From Bench to Bed

**DOI:** 10.3389/fsurg.2021.641370

**Published:** 2021-05-20

**Authors:** Xingdou Mu, Juliang Zhang, Yue Jiang

**Affiliations:** Department of Breast and Thyroid Surgery, Xijing Hospital, Fourth Military Medical University, Xi'an, China

**Keywords:** breast reconstruction, 3D printing, 3D bio-printing, tissue engineering, female breast cancer

## Abstract

Surgical management of breast cancer often results in the absence of the breast. However, existing breast reconstruction methods may not meet the need for a replacement tissue. Tissue engineering with the use of emerging materials offers the promise of generating appropriate replacements. Three-dimensional (3D) printing technology has seen a significantly increased interest and application in medically-related fields in the recent years. This has been especially true in complex medical situations particularly when abnormal or complicated anatomical surgical considerations or precise reconstructive procedures are contemplated. In addition, 3D bio-printing which combines cells with bio-material scaffolds offers an exciting technology with significant applications in the field of tissue engineering. The purpose of this manuscript was to review a number of studies in which 3D printing technology has been used in breast reconstructive surgical procedures, and future directions and applications of 3D bio-printing.

## Introduction

Breast cancer is the most common cancer diagnosed among US women and is second only to lung cancer as a cause of cancer death among women as of 2019. Because ~268,600 (almost six times than DCIS) new cases prove to be an invasive type of breast cancer (1), many women had to choose the removal of the breast, with immediate consideration for a replacement tissue. Although this was satisfactory in many patients, either saline or gel-filled breast implants (2) do carry real risks of complications such as infection, capsular contracture, implant dislocation, or deformities (3, 4). The option of autologous reconstruction can be more texturally natural aesthetically, but it requires a more complex procedure, significant time and expense, and possible muscle weakness or hernia formation at the tissue donor site (5). Tissue engineering intends to address these limitations by combining the 3D printing technology with synthetic or natural structural elements.

Three-dimensional (3D) printing, also known as computer-aided manufacturing (CAM), was based on digital model files using metal powder or plastic and other adhesive materials to construct objects with a computer guided precision, printing layer upon layer. Simplistically, it uses a computer aided design (CAD) program to convert the virtual model of an object into a printable object using an STL (Standard Tessellation Language or STereoLithography) file. The object then gradually and precisely takes shape as each thin layer is added according to the design file, and composed of the desired material for that object in the form of “ink” using the 3D printer. Not only in cases of intraoperative 3D printed models serving as templates, but this technology has extended to implanted scaffolds that have been used to correct defect-specific sites, clearly enhancing patient treatment (6, 7). One such application pertains to an individualized, precise reconstruction of defects in the load bearing axial skeleton (8). However, application of scaffolds employing 3D bio-printing for soft tissues increases the complexity and difficulty dramatically. Different from the hard, anatomically precise skeleton, defects in soft tissue come in a myriad of shapes and sizes that are flexible with a broad spectrum of texture. Materials available for 3D bio-printing that match the wide variety of soft tissue mechanical properties are scarce and do not adequately represent the physical, chemical, and biological complexity and diversity of tissues and organs within the human body (9). Perhaps the most daunting challenge in soft tissue repair beyond anatomical restoration is the prospect of achieving functional restoration. As an example, although considerable efforts have been undertaken, the great challenge to produce a 3D bio-printed, functional tissue-engineered liver scaffold has yet to be produced (10).

Functional restoration in breast reconstruction is a possible exception, as this quality is usually far less critical than achieving optimal cosmetic shape and mechanical properties. The potential impact of exceptional 3D bio-printing scaffolds for breast reconstruction has an immense potential clinical significance. To be integrated into or even replace the current breast reconstruction, such scaffolds would need to meet extraordinarily high qualities of biocompatibility, mechanical properties similar to normal breast tissue, and be biodegradable within a specific period of time.

## Past Work and Current Direction of Tissue Engineering

Since 1986, with the first patent of 3D printing technology—stereolithography, the scope of this innovative technology has undergone explosive expansion in research and application. Following on the introduction of the earliest stereolithography technology (SLA), other techniques of 3D printing technology have been developed such as inkjet-printing, selective laser sintering (SLS), and melt deposition modeling printing methods (Fused Deposition Modeling) (11).

Initially, limited by traditional printing methods and materials, the first applications of 3D printing technology were used in the automotive and aerospace manufacturing industries. However, with continued innovation and evolution of printing methods and materials, 3D printing entered the field of medicine in the early 21st century, principally used in bone and artificial limb implants. In 2000, Thomas Boland of Clemson University first proposed the concept of cell printing which led to its first realization in 2003 (12). Less than two decades later, printed cell scaffolds with a micro-resolution smaller than 100 μm, and a cell survival rate >95% have been reported (13, 14). This level of achievement inspires additional, medically-related 3D printing technology and will almost surely trigger further research in the field of soft tissue repair.

In 2015, the mechanical manufacturing department of Xi'an Jiaotong University developed the melt electrostatic printing technology, combining the advantages of melt electrospinning and 3D printing technology, to produce micro nanofibers (15). This exquisitely fine instrument technology can construct any complex shape of a three-dimensional structure, and of extreme importance, has the unique advantage of simulating the structure of human extracellular matrix. Therefore, it provides an ideal platform for highly precise, 3D printing especially in regard to the overall discipline of medicine.

## Application of 3D Printing Scaffold in Breast Reconstruction

### Application of Tissue Engineering Materials in Breast Reconstruction

In 2011, Melchels et al. (16) first introduced a computer-aided technology to construct 3D models of the breast, laying the foundation for future 3D printing (Table 1). In 2013, Tsuji et al. (17) implanted polypropylene mesh cages into rabbits' bilateral fat pads and injected minced type I collagen sponge into the cage to act as a scaffold. At 6- and 12-months follow-up, study of the removed cages verified that adipose tissue regeneration actually occurred. Although the implant did not match the shape of the breast and was too rigid to replace soft tissue, these results inspired the concept of 3D bio-implants for breast reconstruction.

**Table 1:** Summary of tissue engineering materials in breast reconstruction.

**References**	**Materials**	**Advantages**
Melchels et al. (16)	Computer-aided technology	The concept of 3D models of the breast appeared for the first time, laying the foundation for future 3D printing.
Findlay et al. (18)	Acrylic acid porous chamber	It could produce a quantity of viable breast tissue satisfactory for transplantation.
Tsuji et al. (17)	Polypropylene mesh cages and injected minced type I collagen sponge	Inspired the concept of 3D bio-implants for breast reconstruction.
Chhaya et al. (19)	Multi-layer reticulated polycaprolactone hemispherical scaffold and delayed fat injection	Fat necrosis could be avoided.
Luo et al. (20)	Alg-PDA scaffold	It demonstrated great flexibility and similar elastic modulus to normal breast tissues.
Tytgat et al. (21)	Gel-MA–Car-MA scaffold	Its mechanical properties were comparable with the natural mammary tissue.

Aside from purely cosmetic issues, the size of breast implants poses an additional engineering problem in 3D bioprinting for breast reconstruction. If the implants are too small, they cannot maintain the optimal shape of the breast and will also limit subsequent tissue regeneration. As a solution, Findlay (18) designed a porous chamber, similar to the shape of a female breast, made from acrylic acid, which was implanted into a pig model together with vascularized tissue. This was intended to meet the demand for vascularization of a large quantity of regenerated breast tissue. The results at 6 weeks were successful in producing an implant filled with neovascularized tissue, which was close to the useful volume necessary for human breast reconstruction.

Unfortunately, although this method produced a quantity of viable breast tissue satisfactory for transplantation, the main component was only fibrous tissue, with only a small quantity of fat core inside it. Although the implanted tissue certainly would not collapse after implantation and the appearance could be maintained for a long time, the texture was hard and the cosmetic effect was poor. Additionally, infection after implantation could also occur.

**Figure 1:**
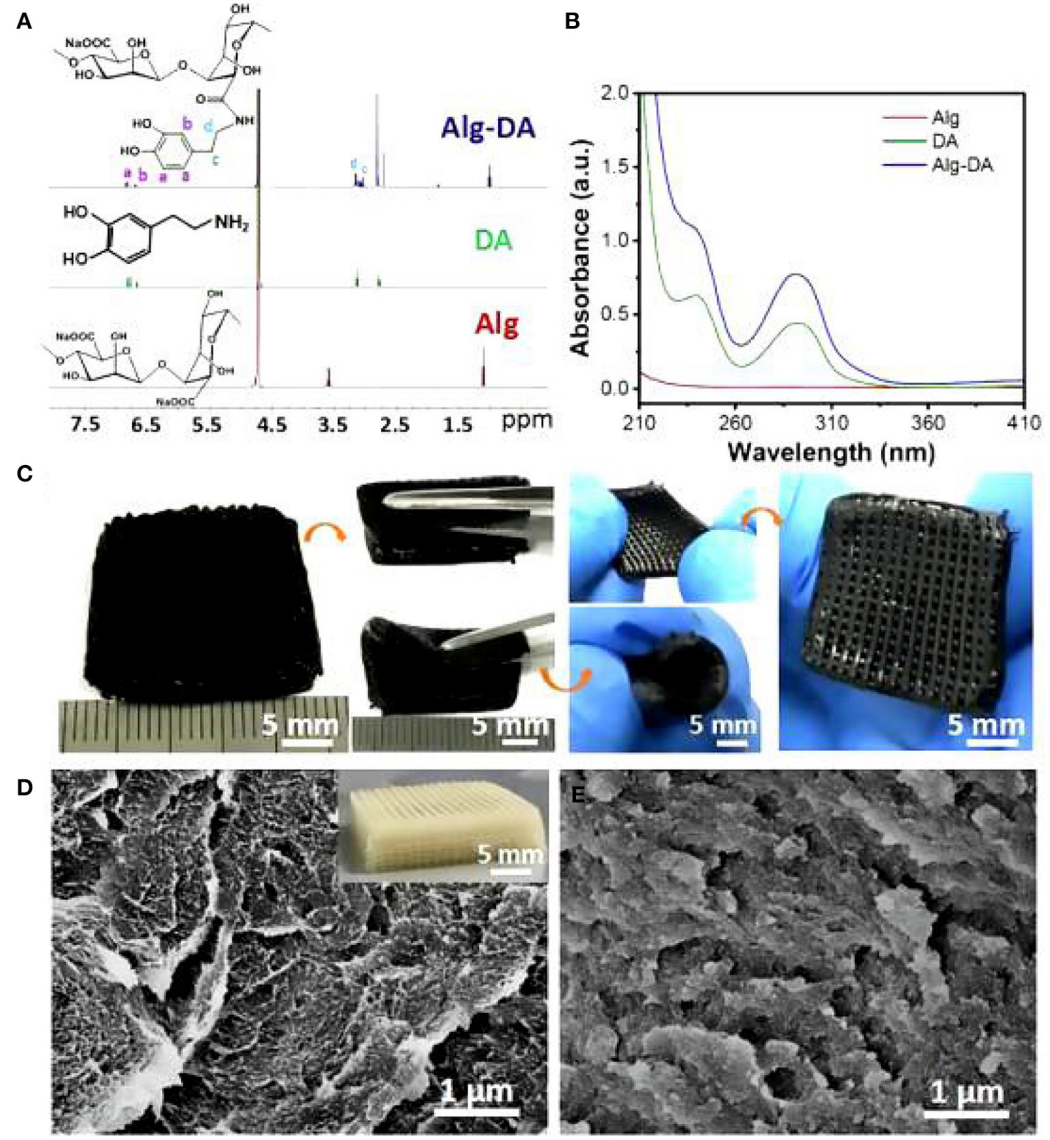
1H NMR spectra **(A)** and UV–Vis absorption **(B)** spectra of Alg, DA, and Alg-DA. Photographs of the 3D-printed Alg-PDA scaffold. The scaffold maintained its original structure without deformation and cracks suffering from bending, rolling, and stretching **(C)**. SEM images in the inside of struts of pure alginate **(D)** and Alg-PDA scaffolds **(E)**.

As noted previously, the criteria for optimal breast reconstruction are quite rigorous. Using a 3D printing technology not only requires the materials to maintain a pleasing cosmetic breast shape, but must also virtually match the human breast in mechanical properties. Therefore, the selection of materials is crucial. In 2016, Chhaya (19) implanted a multi-layer reticulated polycaprolactone hemispherical scaffold into the subglandular pockets of immunocompetent minipigs and injected a small amount of fat at 2 weeks post-implantation. The results showed that fat necrosis could be avoided, and adipose tissue regeneration could be promoted by the delayed fat injection. Polycaprolactone is a kind of bioactive, biodegradable, thermoplastic polymer with excellent biocompatibility and good mechanical properties. The delayed fat injection provided optimal conditions for angiogenesis around the scaffold and guaranteed the survival and subsequent regeneration of adipose tissue. These animal experiments established the basis for a structured scaffold implantation, a technique for stimulation of angiogenesis and optimization of the local microenvironment for various growth factors to play a role in tissue regeneration. Exploring the properties of different materials to perfect breast reconstruction, in 2019, biofunctional scaffolds incorporating dopamine-modified alginate (Alg) and polydopamine (PDA) were fabricated using 3D printing (20). The experimental results showed that the Alg-PDA scaffold demonstrated great flexibility and similar elastic modulus to normal breast tissues (Figure 1).

**Figure 2:**
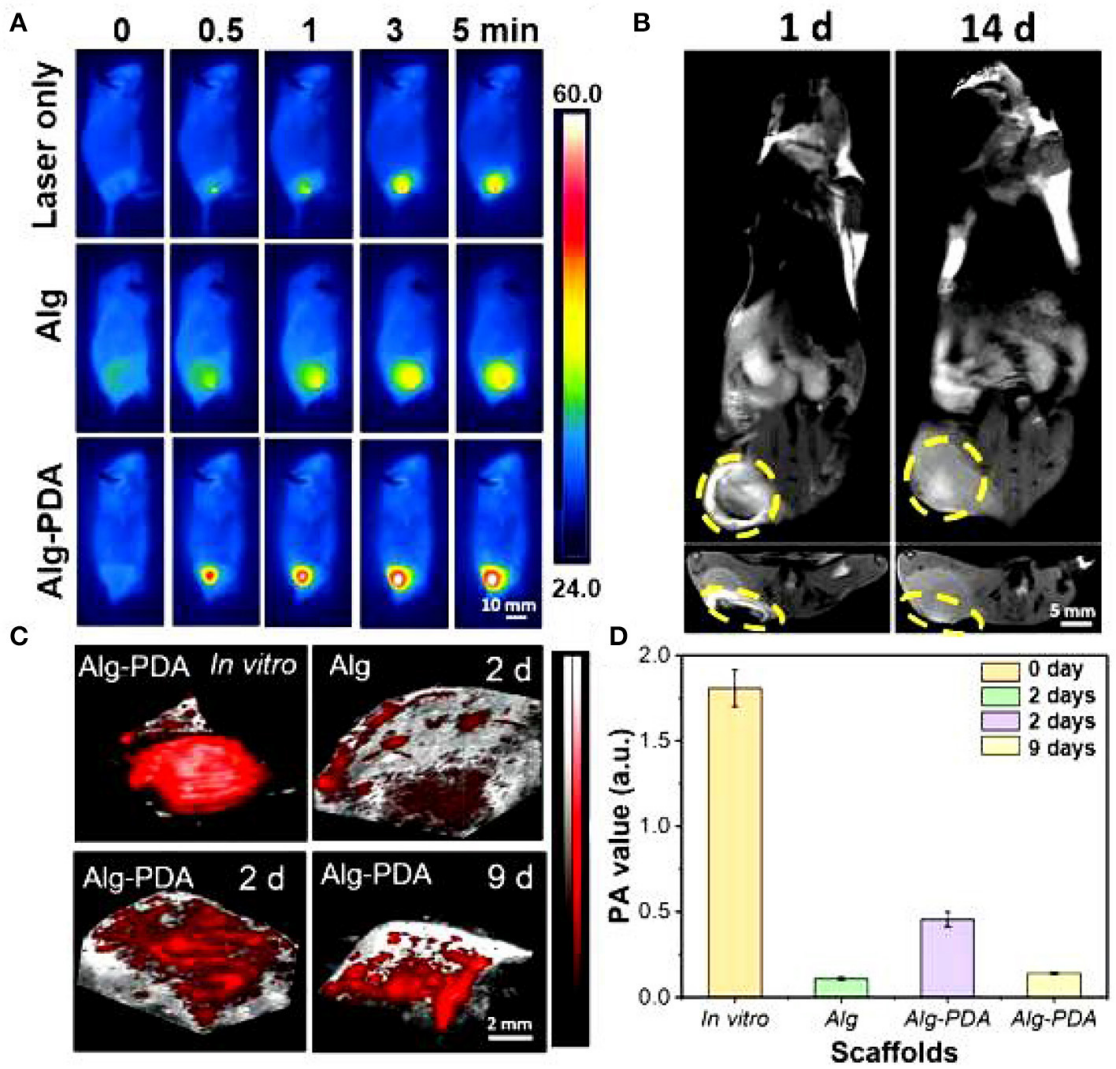
MRI images of the breast cancer region implanted with Alg-PDA scaffold for 1 and 14 days **(A)**. Yellow circles indicate the location of the scaffold **(B)**. Photoacoustic imaging **(C)** and photoacoustic intensity **(D)** of Alg scaffolds and Alg-PDA scaffolds before (*in vitro*) and after implantation at tumor sites of mice for 2 and 9 days.

**Figure 3:**
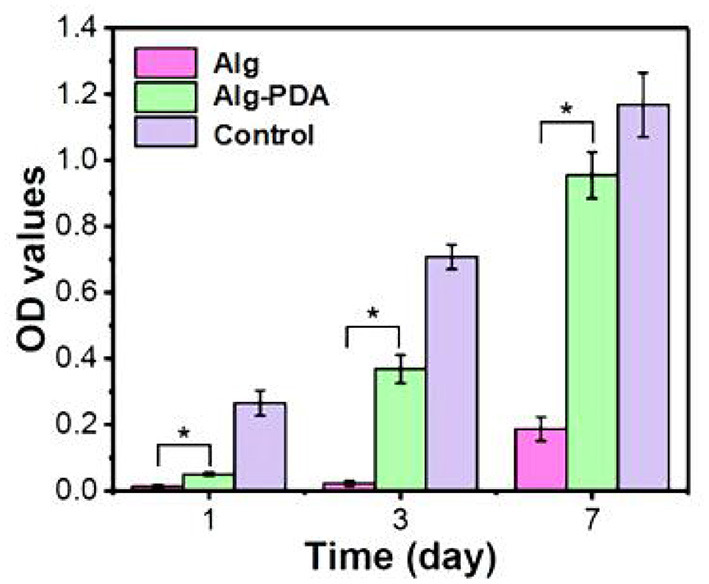
Proliferation of MCF-10A cells seeded on 3D-printed Alg, Alg-PDA, and 48-well plate (control) scaffolds during seven days of culture. © 2019 Acta Materialia Inc. Published by Elsevier Ltd. All rights reserved (20). *indicates statistically significant.

Of particular importance, 14 days following scaffold implantation in mice with a breast cancer, the tumor size of the cancer was significantly reduced. Human breast epithelial cells (MCF-10A) were then implanted on the scaffolds and cultured for seven days. The results showed that the scaffold could support the proliferation of breast epithelial cells (Figures 2, 3).

This kind of PDA scaffold has been used in other biomedical engineering fields, and in the future, we hope to utilize this PDA scaffold in breast reconstruction with the added benefit of reducing the risk of local breast cancer recurrence.

**Figure 4:**
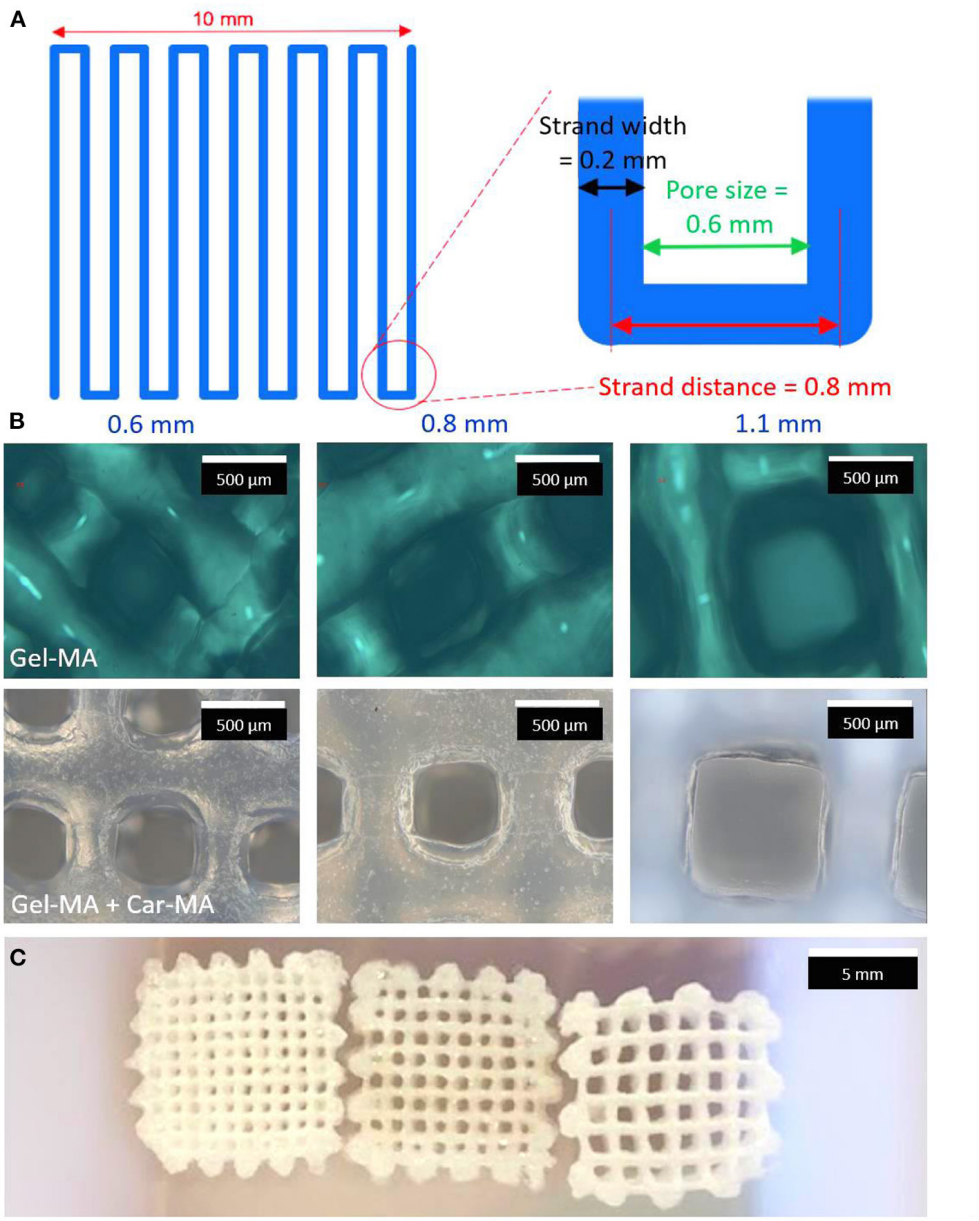
Scheme of a layer of the printed scaffolds **(A)**. Optical microscopy images of Gel-MA (upper panel) and Gel-MA – Car-MA scaffolds (center). The scale bars represent 500 μm **(B)**. Image of freeze-dried Gel-MA – Car-MA scaffolds. The scale bar represents 5 mm **(C)**.

Similarly, Tytgat et al. (21) used an extrusion-based 3D printing to develop scaffolds composed of both methacrylamide-modified gelatin (Gel-MA) and methacrylated κ-carrageenan (Car-MA). *In vitro* experiments showed that this hydrogel scaffold remained stable over time, absorbed large amounts of water, and its mechanical properties were comparable with the natural mammary tissue (Figure 4).

### Clinical Application of 3D Printing Scaffold in Breast Reconstruction

**Figure 5:**
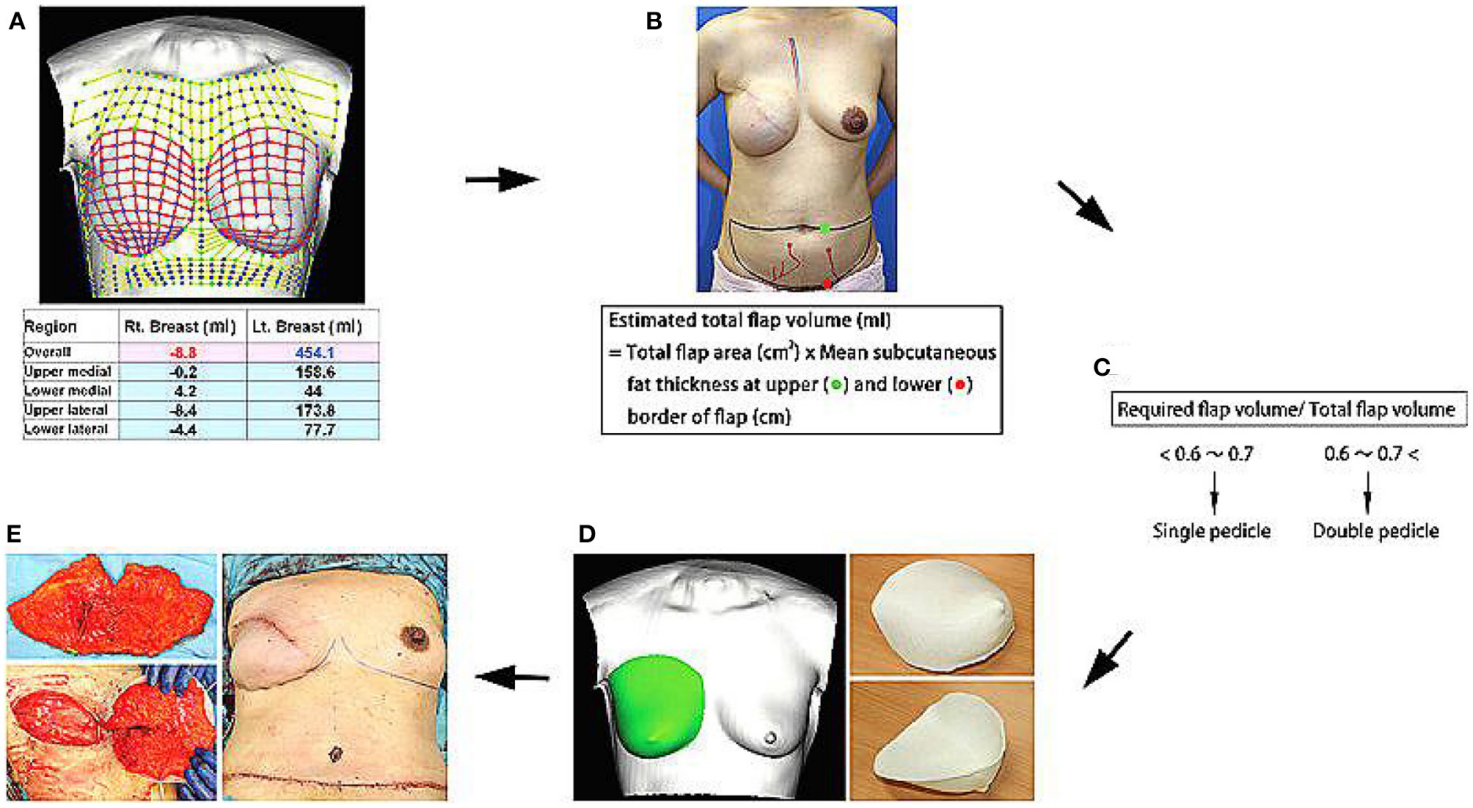
**(A)** Required flap volume was estimated from bilateral breast images using a 3D image data analysis software. **(B,C)** Total flap volume was estimated using the formula shown, and flap type was determined preoperatively. **(D)** Contralateral breast shape was horizontally inverted, and an acrylonitrile–butadiene–styrene copolymer breast mold was created using a personal 3D printer. **(E)** After vascular anastomosis, the de-epithelialized flap was placed in the mold and fixed to shape a symmetric breast. Copyright © 2015, © 2015 American Society of Plastic Surgeons (23).

**Figure 6:**
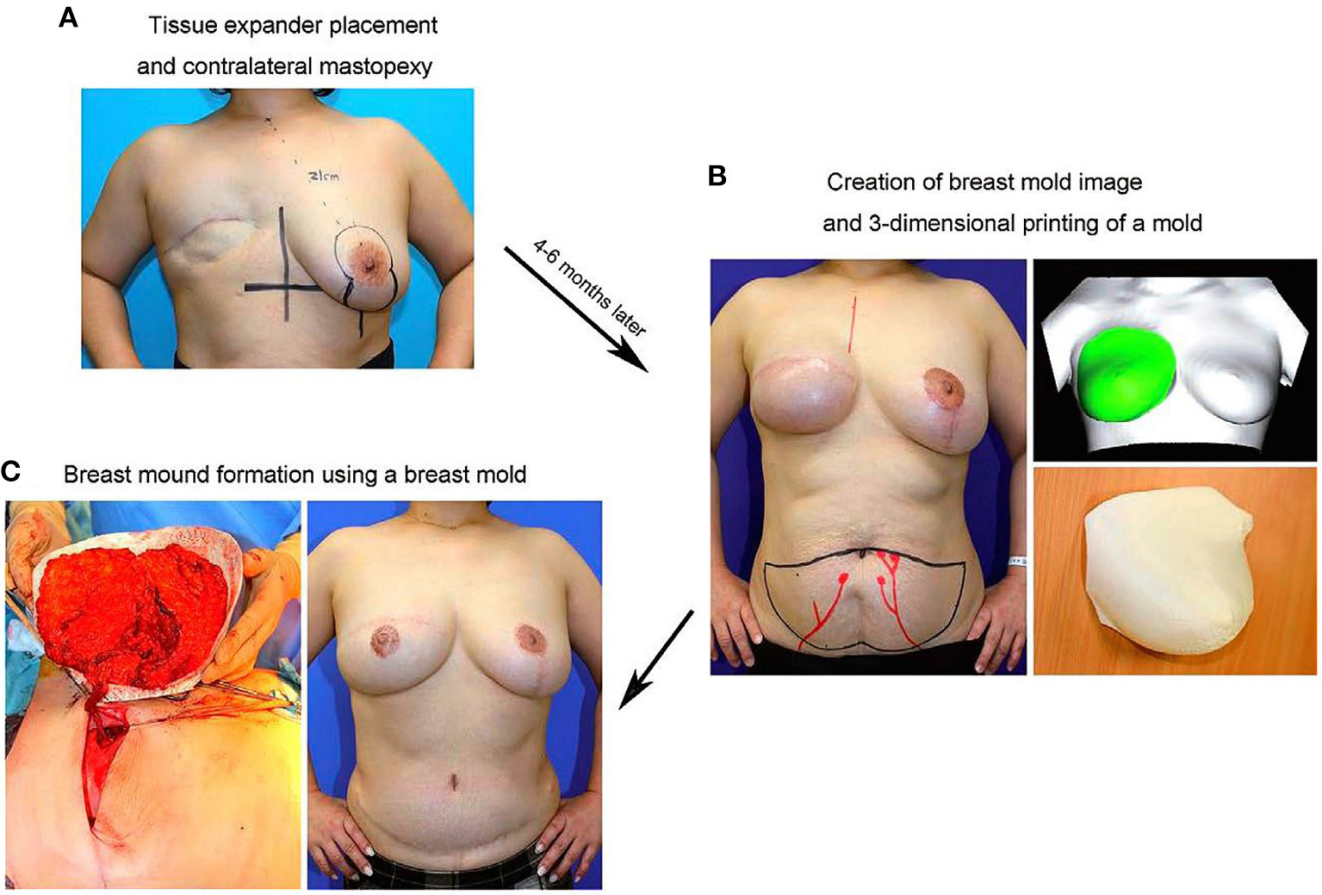
**(A)** In the initial surgery, TE placement in the affected breast and mastopexy of the contralateral breast using the vertical scar technique are performed. **(B)** Four to six months postoperatively, a 3D bilateral breast imaging is performed after confirming that the shape of the contralateral breast is somewhat stabilized, and a 3D-printed breast mold is created based on the mirror image of the shape of the contralateral breast. **(C)** In DIEP flap surgery, the direction of the flap and volume of graft tissue are determined using the breast mold. Copyright © 2017, Copyright © 2017 The Authors. Published by Wolters Kluwer Health, Inc. on behalf of The American Society of Plastic Surgeons (24).

In 2016, a study on tissue engineering for human breast reconstruction was carried out in Australia (22) (Table 2). Morrison designed an acrylic perforated dome-shaped chamber implant with 3 mm holes, ranging in size from 140 to 360 ml. Five female patients, ages 35–49 years, were selected for unilateral breast reconstruction. The specific plan was to implant it with the vascular pedicle fat flap, but it was reoperated to remove the implant 6 months after the initial operation. Analysis of the tissue removed with the implant demonstrated newly formed blood vessels, fibrous tissue, and a portion was adipose tissue. However, the implant material itself was not degradable, the texture was hard, and the resultant cosmetic assessment was poor. Koichi (23) studied bilateral breast reconstruction, utilizing preoperative three-dimensional imaging to estimate the required replacement volume. Then, he used 3D printing technology and polypropylene copolymer as the bioink to print the new breast form. According to the breast volume calculated, a single- or double-pedicle flap was developed to reconstruct the breast in combination with the 3D printed mold (Figure 5). With modest alteration, reconstruction of patients with breast ptosis (24) could also achieve good cosmetic results (Figure 6).

**Table 2:** Summary of 3D printing scaffold in breast reconstruction.

**References**	**Scaffolds**	**Advantages**
Morrison et al. (22)	Acrylic perforated dome-shaped chamber	After 6 months, newly formed blood vessels, fibrous tissue, and a portion was adipose tissue.
Tomita (23, 24)	Polypropylene copolymer breast form	With the 3D printed mold, a single- or double-pedicle flap was developed to reconstruct the breast.
Hummelink (25)	PolyLactic acid breast prosthesis	It used a mirror image of the contralateral breast to design the breast prosthesis by 3D printing.
Juliang (26)	Porous polycaprolactone breast implant	The bioprinting material is a biocompatible and biodegradable polymer.

Stefan (25) took a similar approach, but used a mirror image of the contralateral breast to design the breast prosthesis by 3D printing using PolyLactic Acid as the printing material. Some attempts to utilize this approach to partial breast reconstruction have been disappointing, and some cases would require additional surgery to correct the problem with an obvious negative impact on the physical and mental health of the patients.

Although the emergence of 3D printing technology provides great potential opportunities for breast reconstructive surgeons with a more predictive precision and personalization with regard to the size and shape for the individualized patients, there remain limitations in virtually all aspects including the materials, shape, and structure of the breast prosthesis to be printed. To date, clinical application of 3D printing technology continues to suffer from the same problems as traditional prosthetic reconstruction, such as bilateral breast asymmetry and capsular contraction.

**Figure 7:**
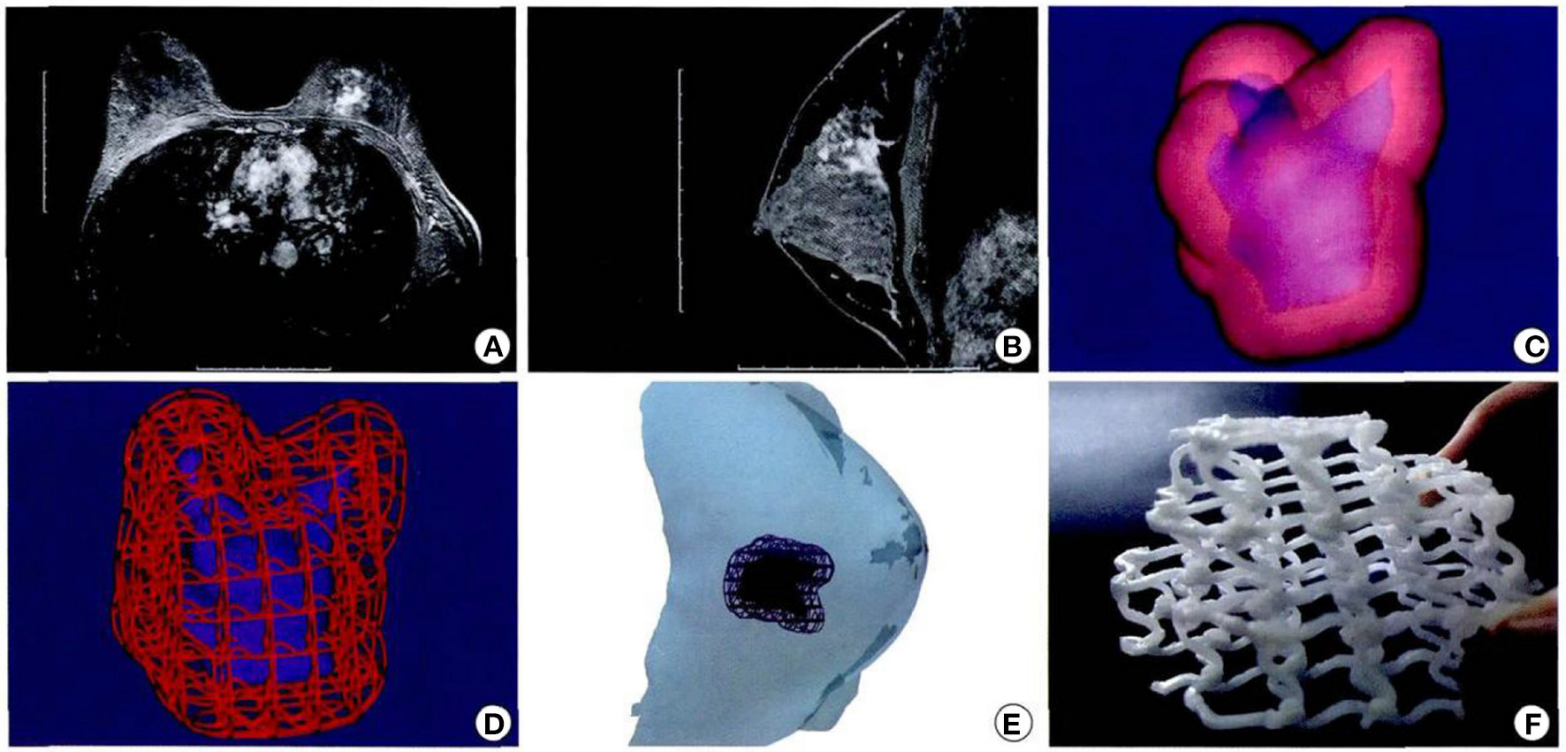
Design and printing of personalized biodegradable implants for patients. **(A,B)** Breast magnetic resonance imaging front view; **(C,D)** Three dimensional images were constructed according to the MRI image; **(E)** Simulated three dimensional images of tumor resection and scaffold implantation in surgery. **(F)** General shape of degradable breast implants (the material is polycaprolactone) and internal structure of degradable breast implants (26).

As an initial introduction to the clinical experience of this technology, a biodegradable breast implant employing 3D printing technology, sized according to a tissue defect from a wide local excision, was undertaken in 2016. Professor Zhang Juliang (26) of Xijing Hospital admitted a 27-year-old female patient with a left breast invasive cancer measuring 4.0 × 3.0 cm. Following the completion of six cycles of neoadjuvant chemotherapy, the cancer had reduced in size to 3.5 × 1.4 × 2.1 cm. The patient was adamantly requesting breast conservation surgery. The decision was made to pursue a wide local excision of the cancer, then, with the use of CAD to measure the resultant breast defect, breast reconstruction would incorporate 3D printed, biodegradable materials. Specifically, the breast MRI plain and enhanced scan data of the surgical defect were used to construct a three-dimensional image to precisely define the size and shape of the breast implant. The bioprinting material was the same as previously described, polycaprolactone, a biocompatible and biodegradable polymer. The preset deformation and degradation time was expected to be 2 years. The 3D biomaterial printer, developed independently by the State Key Laboratory of mechanical manufacturing system engineering of Xi'an Jiaotong University, was used to print the personalized porous breast implant, ideal for the needs of the patient (Figures 7A–F).

**Figure 8:**
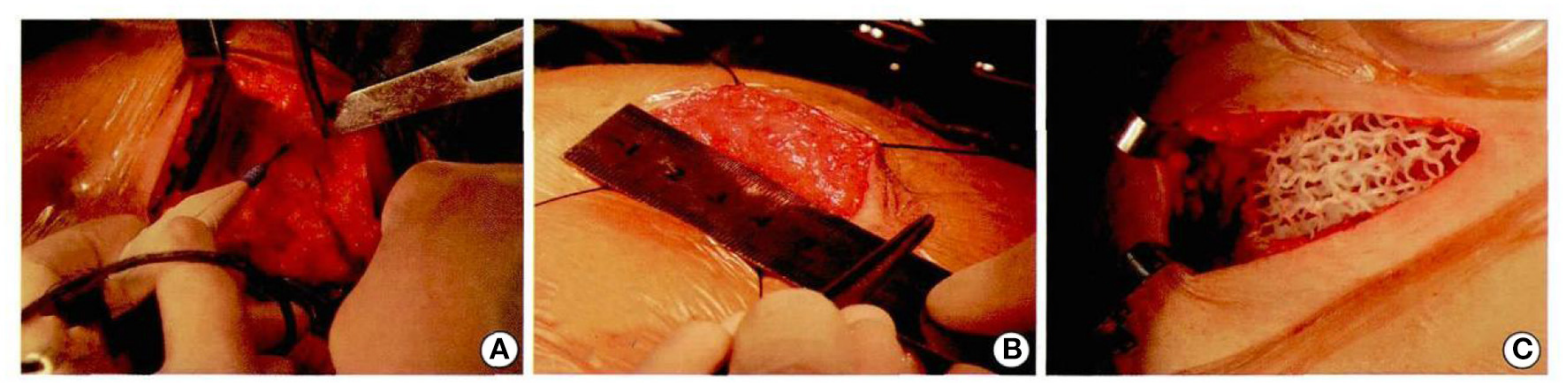
Patients underwent computer-assisted 3D printing of degradable materials for breast reconstruction **(A–C)**.

**Figure 9:**
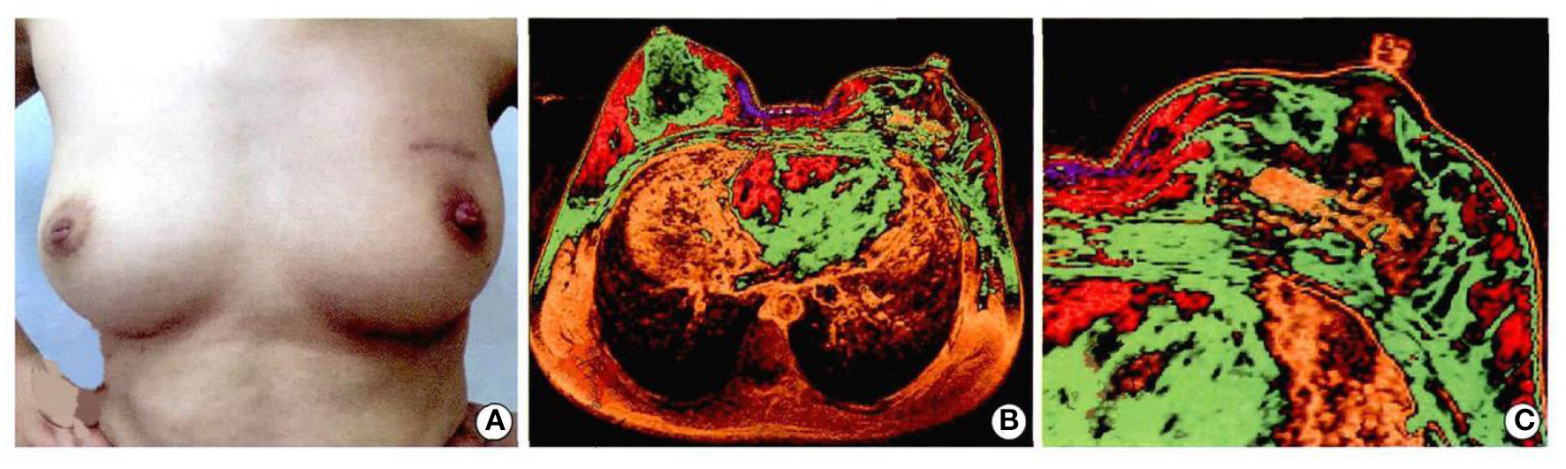
Patients with breast cancer underwent left breast segment resection + 3D printing implant + axillary lymph nodes dissection. The breast appearance and follow-up of 9 months were as follows: **(A)** figure: cosmetic effect of breast; **(B)** magnetic resonance imaging of breast; **(C)** magnetic resonance imaging of implant. Written informed consent was obtained from the patient for the publication of any potentially identifiable images or data included in this article (26).

The whole operation was performed under aseptic conditions (Figures 8A–C). Follow-up 9 months later was particularly encouraging, with a good cosmetic appearance, and the MRI showed that the implant had a good compatibility with the patient's own autogenous tissue. There was an abundant vascularity and granulation tissue throughout the implant, especially through the holes in the scaffold, and the appearance of new soft tissue (Figures 9A–C). From an oncologic perspective, with the follow-up extended to the end of December 2017, there was no recurrence or evidence of metastasis.

Subsequently, the team has carried out breast reconstruction with 3D printed degradable implants for more than 15 patients. Postoperative follow-up has continued to show good cosmetic results with no significant complications. The new availability of 3D printed, degradable breast implants has provided a welcome solution to the dilemma of a relatively large breast cancer that necessitates a large volume local resection resulting with a proportionately large breast defect. Patients' strong preference for breast conserving surgery can now be accommodated without a serious cosmetic compromise, permitting a clearly enhanced quality of life of these patients. The advantages of this novel technique are many: the scaffold can be customized in size and shape according to the individualized needs of the patient; the degradation time and scaffold strength can be adjusted; and the mesh pore structure of the implant provides access for fat injection at the implantation site as desired after the operation. Moreover, this kind of breast scaffold can be “printed” relatively simply and at a low cost, which can not only meet the needs of different patients after breast conserving surgery, but also be suitable for large-scale clinical implementation.

### Progress in 3D Cell Printing Technology

While the current biomaterials for bioprinted breast reconstruction have facilitated advances in constructing an individualized shape and size of the breast, further innovations can be expected in tissue matching, improved cosmesis, stimulation of adipose tissue regeneration, and potentially even in recovery of the true biologic breast function. Although the demand for a fully functional breast is not urgent, it is likely to surface as a hot topic in the future of 3D printing technology.

In 2015, Chhaya (27) seeded breast-shaped scaffolds with human umbilical cord perivascular cells, then subsequently seeded the scaffolds with human umbilical vein endothelial cells after 6 weeks of culturing. They implanted these composite cells impregnated scaffolds subcutaneously into athymic nude rats for 24 weeks. The increase in new adipose tissue was dramatic: the ratio of adipose to overall tissue area increased from 37.17 to 62.30% between weeks 5 and 15 (*p* < 0.01), and further increased to 81.2% at week 24 (*p* < 0.01). Simultaneously, the inoculated endothelial cells transformed into functional capillary networks. This innovative process laid the foundation for synthesizing implantation of the combined scaffold and cell components. This process not only accelerated tissue repair, but also induced adipose tissue regeneration, far superior to the former fibrous scar tissue. This was rapidly followed by a report in 2018 by Rossi (28) of a scaffold that was decorated with extracellular matrix (ECM) deposited by human adipose derived stromal cells (hADSCs), then implanted subcutaneously in athymic nude mice. The results confirmed that scaffolds mixed with cell matrix had the capability to induce fat regeneration. In 2019, Tytgat et al. (29) inoculated scaffolds composed of two hydrogels, Gel-MA, and Gel-MA-Car-MA, with adipose derived stem cells. The results demonstrated that ADSCs could survive at least 2 weeks on both scaffolds and the survival rate of Gel-MA hydrogel scaffolds was >90%. Furthermore, ADSCs could differentiate into adipogenic lineage on both scaffolds. Similar to mesenchymal stem cells, ADSCs possess a multilineage potential, including the ability to differentiate into adipocytes, and they can self-renew, making them ideal for adipose tissue regeneration and angiogenesis. Their main advantage compared to mesenchymal stem cells is that they can be easily and repeatedly harvested using minimally invasive techniques with a low morbidity, making these cells ideal for application in regenerative therapies (30–32).

Notably, these studies confirmed that when active cell components or cell matrix participated in tissue repair, they had the ability to induce and even accelerate fat regeneration. The challenge remains, however, whether 3D printed cell scaffolds can be used to regenerate breast glandular tissue. In 2016, Ethan (33) isolated primary human breast epithelial cells from patient reduction mammoplasty tissues and seeded them into 3D hydrogels. Perhaps surprisingly, the results showed that these cells could rapidly self-organize in the absence of stromal cells, and within 2 weeks, they could expand to form mature mammary tissues. The mature tissues contained luminal, basal, and stem cells and also exhibited the complex ductal and lobular morphologies normally observed in the female human breast. When treated with estrogen and progesterone and with the further addition of prolactin, it could produce lipid droplets, indicating that they were responding to the hormones. The excellent breast tissue regeneration capability of this 3D cell printing scaffold provided a new inspiration for future breast reconstruction. Not only was it possible to induce a new production of adipose tissue, but also, the regeneration of a normal breast tissue with all elements present. Although typically, the bioprinting of breast scaffolds has been limited to animal experiments, the Texas Department of biomedical engineering has teamed up with Tevido Biodevices to develop 3D bioprinted breast implants (34). Similarly, researchers at Queensland University of Technology have been investigating bioabsorbable 3D printing scaffolds and plan to use them clinically for breast reconstruction in the next few years (35).

## Discussion

The studies reviewed here show that the promise of 3D printing is becoming fulfilled with regard to breast reconstruction. However, considerable work remains to verify the technique to enable a wider clinical application. Melchels et al. introduced the possibility of 3D printing for breast reconstruction and its favorable outcome stimulated subsequent research (16–18). The most important attribute of 3D printing is that it has a wide use, clinically, to solve practical problems. Zhang has shown that it will enable the personalization of bioprinted tissues and large-scale clinical applications will become a reality in the future (26). Chhaya et al. sought to stimulate regeneration by delayed lipoaspirate injection to the implant site (19). Clearly, however, newly injected adipose tissue lacks vasculature that often results in volume loss over time (5, 29). Therefore, newly developed biomaterial inks must keep fat cells alive. Pati et al. have shown that decellularized extracellular matrix (dECM) bioink can provide an optimized microenvironment to induce adipocyte differentiation from ASCs, and they have “printed” a cell-laden structure using human adipose-derived stem cells (hASCs) (36, 37). Yoshimura et al. pioneered a novel technique known as cell-assisted lipotransfer (CAL), in which autologous fat grafts are enriched with autologous ASCs (38). These techniques demonstrate the exciting potential of fat stem cells for breast reconstruction, recognizing that their safety specifically with regard to the potential breast cancer recurrence needs to be further investigated. Eterno et al. found that ASCs contribute to the metastasis and proliferation of c-Met expressing breast cancer cells (39), and Sakurai et al. suggested that cytokine production by ASCs had the potential to stimulate breast carcinoma cell growth by the upregulation of S100A7 expression (40).

## Conclusion

Overall, the functional requirement of breast tissue is not high, mainly aesthetic, and tissue matching, which is suitable for breast reconstruction by 3D printing technology. With the development of new materials and cell printing technology, scaffolds which can perfectly repair defects have broad application prospects, so as to achieve a personalized reconstruction and repair.

## Author Contributions

JZ and XM: design, collection of data, manuscript, editing, approval of final version, and accountability. YJ: collection of data. All authors contributed to the article and approved the submitted version.

## Conflict of Interest

The authors declare that the research was conducted in the absence of any commercial or financial relationships that could be construed as a potential conflict of interest.
